# Supplementary figures and images for: T-cell activation and senescence in asymptomatic HIV/Leishmania infantum co-infection
Source: PLoS Negl Trop Dis. 2025 Mar 17;19(3):e0012848. doi: 10.1371/journal.pntd.0012848 (PMC11964262; doi:10.1371/journal.pntd.0012848)

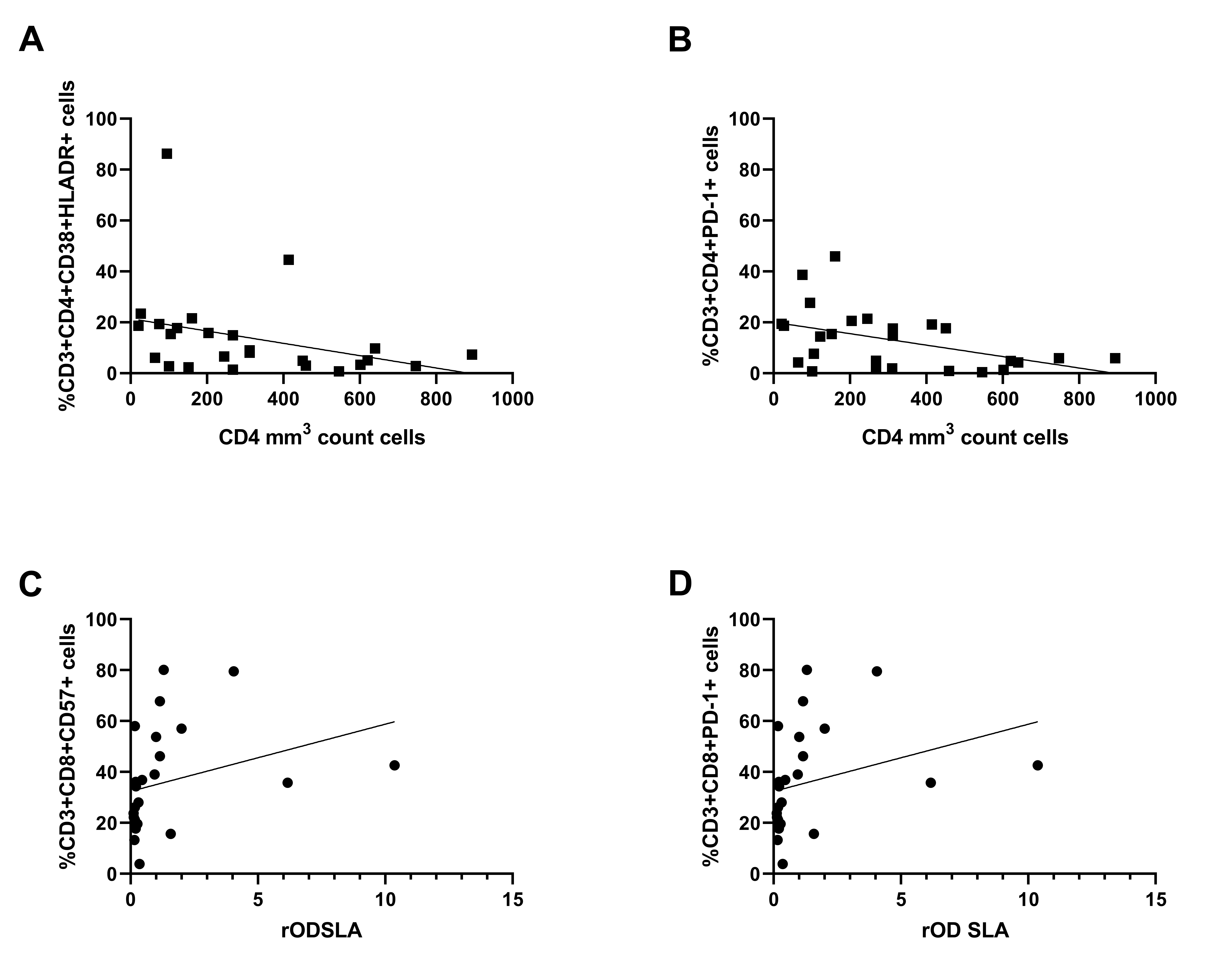

Supplement: S1 Fig — A. Activation in CD4+; B. Exhaustion in CD4+, C. Senescence in CD8+ and D. Exhaustion in CD8+. (TIF) [file pntd.0012848.s001.tif]

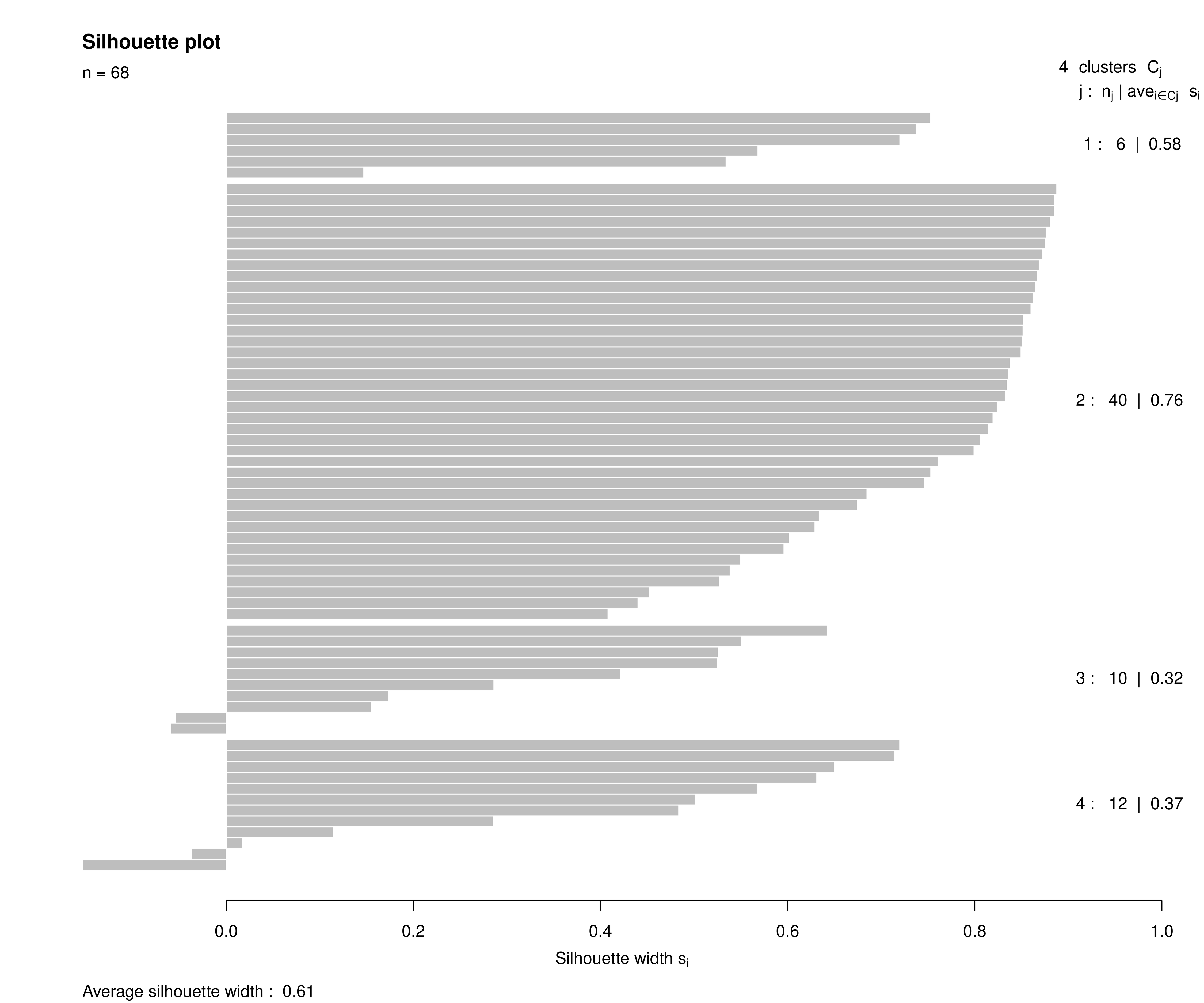

Supplement: S2 Fig — For clusters 1, 2, 3, and 4, the number of subjects (the average silhouette width) in each cluster are 6 (0.58), 40 (0.76), 10 (0.32) and 12 (0.37), respectively. The best value of the average silhouette width was 0.61. s_i is the silhouette width (or score) and J is the cluster name and 1, (n_j) is number of individuals in the cluster and ave_iEC_i is the mean of the silhouette width in cluster. Each cluster has a j, n-j and ave_iEC_i. (TIF) [file pntd.0012848.s002.tif]
