# Supplementary material for: T-cell activation and senescence in asymptomatic HIV/Leishmania infantum co-infection
Source: PLoS Negl Trop Dis. 2025 Mar 17;19(3):e0012848. doi: 10.1371/journal.pntd.0012848 (PMC11964262; doi:10.1371/journal.pntd.0012848)
Supplement: S1 Table — (DOCX) [file pntd.0012848.s003.docx]

| **Table S1. Pairwise comparisons of the marginal mean of the OD_SLA considering a Generalized Linear Model fit** | | | | |
| --- | --- | --- | --- | --- |
| **Group comparisons** | Estimate | Std. Error | t.ratio | p-value |
| HEALTHY - (AIDS/VL) | -1.7776 | 0.559 | -3.180 | 0.0369 |
| HEALTHY - (Asympt HIV/VL) | -1.0827 | 0.425 | -2.548 | 0.1632 |
| HEALTHY - (DTH+) | -1.6629 | 0.590 | -2.820 | 0.0900 |
| HEALTHY - HIV | -0.0159 | 0.132 | -0.120 | 1.0000 |
| HEALTHY - RECOVERED VL | -3.6024 | 1.326 | -2.718 | 0.1134 |
| HEALTHY - VL | -5.2797 | 1.636 | -3.227 | 0.0326 |
| (AIDS/VL) - (Asympt HIV/VL) | 0.6949 | 0.685 | 1.015 | 0.9484 |
| (AIDS/VL) - (DTH+) | 0.1146 | 0.798 | 0.144 | 1.0000 |
| (AIDS/VL) - HIV | 1.7617 | 0.553 | 3.186 | 0.0363 |
| (AIDS/VL) - RECOVERED VL | -1.8248 | 1.430 | -1.276 | 0.8598 |
| (AIDS/VL) - VL | -3.5022 | 1.722 | -2.034 | 0.4061 |
| (Asympt HIV/VL) - (DTH+) | -0.5802 | 0.710 | -0.817 | 0.9822 |
| (Asympt HIV/VL) - HIV | 1.0668 | 0.417 | 2.559 | 0.1595 |
| (Asympt HIV/VL) - RECOVERED VL | -2.5196 | 1.383 | -1.822 | 0.5399 |
| (Asympt HIV/VL) - VL | -4.1970 | 1.683 | -2.494 | 0.1821 |
| (DTH+) - HIV | 1.6470 | 0.584 | 2.820 | 0.0898 |
| (DTH+) - RECOVERED VL | -1.9394 | 1.442 | -1.345 | 0.8278 |
| (DTH+) - VL | -3.6168 | 1.732 | -2.088 | 0.3742 |
| HIV - RECOVERED VL | -3.5865 | 1.323 | -2.711 | 0.1151 |
| **HIV - VL** | **-5.2638** | **1.634** | **-3.222** | 0.0331 |
| RECOVERED VL - VL | -1.6774 | 2.100 | -0.799 | 0.9841 |
