# Supplementary material for: T-cell activation and senescence in asymptomatic HIV/Leishmania infantum co-infection
Source: PLoS Negl Trop Dis. 2025 Mar 17;19(3):e0012848. doi: 10.1371/journal.pntd.0012848 (PMC11964262; doi:10.1371/journal.pntd.0012848)
Supplement: S2 Table — (DOCX) [file pntd.0012848.s004.docx]

| **Table S2. Pairwise comparisons of the marginal means of CD4+ Tand CD8+ T cell counts via Generalized Linear Mode fit** | | | | |
| --- | --- | --- | --- | --- |
|  | CD4 |  |  |  |
| Group comparison | Estimate | Std. Error | z-value | p-value |
| HIV - (AIDS/VL) | 495.7 | 100 | 4.938 | <0.0001 |
| HIV - (Asympt HIV/VL) | 74.2 | 149 | 0.498 | 0.8724 |
| (AIDS/VL) - (Asympt HIV/VL) | -421.5 | 113 | -3.746 | 0.0005 |
|  | CD8 |  |  |  |
| HIV - (AIDS/VL) | 426 | 270 | 1.577 | 0.2554 |
| HIV - (Asympt HIV/VL) | -528 | 481 | -1.097 | 0.5162 |
| (AIDS/VL) - (Asympt HIV/VL) | -953 | 454 | -2.098 | 0.0903 |
