# Supplementary material for: T-cell activation and senescence in asymptomatic HIV/Leishmania infantum co-infection
Source: PLoS Negl Trop Dis. 2025 Mar 17;19(3):e0012848. doi: 10.1371/journal.pntd.0012848 (PMC11964262; doi:10.1371/journal.pntd.0012848)
Supplement: S3 Table — (DOCX) [file pntd.0012848.s005.docx]

**Table S3. Proportions of subjects with makers of immune activation and senescence, PD1+ in T cell subsets**

|  | **CD3^+^CD4^+^ T cell** | | |  | **CD3^+^CD8^+^ T cell** | | |
| --- | --- | --- | --- | --- | --- | --- | --- |
|  | **CD38^+^HLA-DR^+^** | **CD57+** | **PD1+** |  | **CD38^+^HLA-DR^+^** | **CD57+** | **PD1+** |
| UC  (n=6) | 2.0%  (0.8-13.7) | 12.87%  (1.27-37.0) | 8.65%  (3.9-12.9) |  | 2.42%  (0.54-3.58) | 39.4%  (15.9-60.1) | 0.04%  (0.0-0.55) |
| HIV  (n=16) | 2.52%  (0.78-17.7) | 4.1%  (0.73-52.2) | 5.1%  (0.18-15.4) |  | 4.19%  (2.14-25.1) | 39.1%  (17.1-69.6) | 2.52%  (0.01-58.0) |
| Asympt HIV/Leish (n=10) | 6.89%  (3.31-15.8) | 15.65%  (2.6-66.7) | 5.37%  (1.3-21.4) |  | 24.55%  (6.65-50.4) | 62.8%  (42.6-88.0) | 42.6%  (15.7-80.1) |
| AIDS/VL  (n=14) | 18.89%  (0.74-86.2) | 10.88%  (0.2-40.0) | 18.0  (0.4-40.2) |  | 36.85%  (14.7-96.9) | 54.6%  (21.3-72.4) | 21.45%  (6.2-57.0) |
| VL  (n=8) | 14.2%  (0.85-31.7) | 5.81%  (1.2-28.7) | 11.7%  (0.7-45.9) |  | 54.05%  (2.08-65.0) | 49.7%  (11.1-59.1) | 15.15%  (4.32-55.7) |
| Recovered VL (n=9) | 2.7%  (1.34-7.1) | 6.82%  (3.0-47.9) | 18.95%  (5.93-40.1) |  | 5.41%  (2.77-12.5) | 43.6%  (11.2-71.2) | 1.55%  (0.28-12.2) |
| DTH+  (n=6) | 0.39%  (0.02-1.83) | 8.3%  (0.71-17.6) | 4.02%  (1.82-30.9) |  | 0.08%  (0.0-7.42) | 35.1%  (12.0-59.6) | 0.24%  (0.0-1.69) |

Results were expressed Median (Min-Max)
