# Supplementary material for: T-cell activation and senescence in asymptomatic HIV/Leishmania infantum co-infection
Source: PLoS Negl Trop Dis. 2025 Mar 17;19(3):e0012848. doi: 10.1371/journal.pntd.0012848 (PMC11964262; doi:10.1371/journal.pntd.0012848)
Supplement: S4 Table — (DOCX) [file pntd.0012848.s006.docx]

| **Table S4. Pairwise comparisons of the marginal means of %** CD3+CD4+CD38+HLA-DR+  **via GLM fit** | | | | | |
| --- | --- | --- | --- | --- | --- |
|  | Estimate | Std. Error | t-value | p-value |  |
| HEALTHY - (AIDS/VL) | -18.343 | 5.845 | -3.138 | 0.0394 |  |
| HEALTHY - (Asympt HIV/VL) | -3.603 | 2.904 | -1.241 | 0.8752 |  |
| HEALTHY - (DTH+) | 3.719 | 1.716 | 2.168 | 0.3278 |  |
| HEALTHY - HIV | 0.562 | 1.926 | 0.292 | 0.9999 |  |
| HEALTHY - RECOVERED VL | 1.355 | 2.073 | 0.654 | 0.9946 |  |
| HEALTHY - VL | -9.681 | 4.902 | -1.975 | 0.4407 |  |
| (AIDS/VL) - (Asympt HIV/VL) | 14.740 | 6.069 | 2.429 | 0.2044 |  |
| **(AIDS/VL) - (DTH+)** | **22.062** | **5.599** | **3.941** | **0.0037** |  |
| **(AIDS/VL) - HIV** | **18.905** | **5.667** | **3.336** | **0.0230** |  |
| (AIDS/VL) - RECOVERED VL | 19.698 | 5.718 | 3.445 | 0.0169 |  |
| (AIDS/VL) - VL | 8.662 | 7.241 | 1.196 | 0.8929 |  |
| (Asympt HIV/VL) - (DTH+) | 7.322 | 2.370 | 3.090 | 0.0448 |  |
| (Asympt HIV/VL) - HIV | 4.165 | 2.526 | 1.649 | 0.6518 |  |
| (Asympt HIV/VL) - RECOVERED VL | 4.958 | 2.640 | 1.878 | 0.5023 |  |
| (Asympt HIV/VL) - VL | -6.077 | 5.167 | -1.176 | 0.9004 |  |
| (DTH+) - HIV | -3.157 | 0.945 | -3.341 | 0.0227 |  |
| (DTH+) - RECOVERED VL | -2.364 | 1.218 | -1.941 | 0.4617 |  |
| (DTH+) - VL | -13.400 | 4.606 | -2.909 | 0.0706 |  |
| HIV - RECOVERED VL | 0.793 | 1.500 | 0.529 | 0.9983 |  |
| HIV - VL | -10.242 | 4.688 | -2.185 | 0.3186 |  |
| RECOVERED VL - VL | -11.036 | 4.751 | -2.323 | 0.2498 |  |
