# Supplementary material for: T-cell activation and senescence in asymptomatic HIV/Leishmania infantum co-infection
Source: PLoS Negl Trop Dis. 2025 Mar 17;19(3):e0012848. doi: 10.1371/journal.pntd.0012848 (PMC11964262; doi:10.1371/journal.pntd.0012848)
Supplement: S5 Table — (DOCX) [file pntd.0012848.s007.docx]

| **Table S5. Pairwise comparisons of the marginal means of the percent of** CD3+CD8+CD38+HLA-DR+ **via GLM fit** | | | | |  |
| --- | --- | --- | --- | --- | --- |
|  | Estimate | Std. Error | t-value | p-value | |
| HEALTHY - (AIDS/VL) | -38.3545 | 8.264 | -4.641 | 0.0004 | |
| HEALTHY - (Asympt HIV/VL) | -22.4247 | 5.949 | -3.769 | 0.0065 | |
| HEALTHY - (DTH+) | -0.0317 | 0.885 | -0.036 | 1.0000 | |
| HEALTHY - HIV | -4.5292 | 1.432 | -3.162 | 0.0372 | |
| HEALTHY - RECOVERED VL | -4.0583 | 2.037 | -1.992 | 0.4300 | |
| HEALTHY - VL | -40.9717 | 11.620 | -3.526 | 0.0135 | |
| (AIDS/VL) - (Asympt HIV/VL) | 15.9299 | 10.139 | 1.571 | 0.7008 | |
| (AIDS/VL) - (DTH+) | 38.3229 | 8.258 | 4.641 | 0.0004 | |
| (AIDS/VL) - HIV | 33.8254 | 8.334 | 4.059 | 0.0026 | |
| (AIDS/VL) - RECOVERED VL | 34.2962 | 8.459 | 4.054 | 0.0026 | |
| (AIDS/VL) - VL | -2.6171 | 14.228 | -0.184 | 1.0000 | |
| (Asympt HIV/VL) - (DTH+) | 22.3930 | 5.941 | 3.769 | 0.0065 | |
| (Asympt HIV/VL) - HIV | 17.8955 | 6.047 | 2.960 | 0.0626 | |
| (Asympt HIV/VL) - RECOVERED VL | 18.3663 | 6.218 | 2.954 | 0.0635 | |
| (Asympt HIV/VL) - VL | -18.5470 | 13.021 | -1.424 | 0.7866 | |
| (DTH+) - HIV | -4.4975 | 1.397 | -3.220 | 0.0318 | |
| (DTH+) - RECOVERED VL | -4.0267 | 2.012 | -2.001 | 0.4246 | |
| (DTH+) - VL | -40.9400 | 11.616 | -3.525 | 0.0135 | |
| HIV - RECOVERED VL | 0.4708 | 2.306 | 0.204 | 1.0000 | |
| HIV - VL | -36.4425 | 11.670 | -3.123 | 0.0413 | |
| RECOVERED VL - VL | -36.9133 | 11.760 | -3.139 | 0.0396 | |
