# Supplementary material for: T-cell activation and senescence in asymptomatic HIV/Leishmania infantum co-infection
Source: PLoS Negl Trop Dis. 2025 Mar 17;19(3):e0012848. doi: 10.1371/journal.pntd.0012848 (PMC11964262; doi:10.1371/journal.pntd.0012848)
Supplement: S6 Table — (DOCX) [file pntd.0012848.s008.docx]

| **Table S6. Pairwise comparisons of the marginal means of the percent of CD3+CD4+CD57+ proportion via beta regression model fit** | | | | |
| --- | --- | --- | --- | --- |
|  | Estimate | Std. Error | z-value | p-value |
| HEALTHY - (AIDS/VL) | -0.01235 | 0.0503 | -0.246 | 1.0000 |
| HEALTHY - (Asympt HIV/VL) | -0.04676 | 0.0564 | -0.829 | 0.9820 |
| HEALTHY - (DTH+) | 0.03530 | 0.0496 | 0.711 | 0.9920 |
| HEALTHY - HIV | 0.03849 | 0.0460 | 0.837 | 0.9811 |
| HEALTHY - RECOVERED VL | -0.01019 | 0.0599 | -0.170 | 1.0000 |
| HEALTHY - VL | 0.02583 | 0.0517 | 0.499 | 0.9989 |
| (AIDS/VL) - (Asympt HIV/VL) | -0.03441 | 0.0478 | -0.719 | 0.9915 |
| (AIDS/VL) - (DTH+) | 0.04765 | 0.0396 | 1.202 | 0.8937 |
| (AIDS/VL) - HIV | 0.05084 | 0.0350 | 1.454 | 0.7721 |
| (AIDS/VL) - RECOVERED VL | 0.00216 | 0.0519 | 0.042 | 1.0000 |
| (AIDS/VL) - VL | 0.03818 | 0.0422 | 0.904 | 0.9720 |
| (Asympt HIV/VL) - (DTH+) | 0.08206 | 0.0471 | 1.741 | 0.5884 |
| (Asympt HIV/VL) - HIV | 0.08525 | 0.0433 | 1.970 | 0.4342 |
| (Asympt HIV/VL) - RECOVERED VL | 0.03657 | 0.0578 | 0.632 | 0.9958 |
| (Asympt HIV/VL) - VL | 0.07258 | 0.0493 | 1.471 | 0.7620 |
| (DTH+) - HIV | 0.00319 | 0.0340 | 0.094 | 1.0000 |
| (DTH+) - RECOVERED VL | -0.04550 | 0.0513 | -0.887 | 0.9745 |
| (DTH+) - VL | -0.00948 | 0.0414 | -0.229 | 1.0000 |
| HIV - RECOVERED VL | -0.04869 | 0.0477 | -1.020 | 0.9497 |
| HIV - VL | -0.01267 | 0.0370 | -0.343 | 0.9999 |
| RECOVERED VL - VL | 0.03602 | 0.0533 | 0.676 | 0.9939 |
