# Supplementary material for: T-cell activation and senescence in asymptomatic HIV/Leishmania infantum co-infection
Source: PLoS Negl Trop Dis. 2025 Mar 17;19(3):e0012848. doi: 10.1371/journal.pntd.0012848 (PMC11964262; doi:10.1371/journal.pntd.0012848)
Supplement: S7 Table — (DOCX) [file pntd.0012848.s009.docx]

| **Table S7. Pairwise comparisons of the marginal means of the percent of CD3+CD8+CD57+ proportion via beta regression model fit** | | | | |
| --- | --- | --- | --- | --- |
|  | Estimate | Std. Error | z-value | p-value |
| HEALTHY - (AIDS/VL) | -0.09817 | 0.0764 | -1.286 | 0.8588 |
| HEALTHY - (Asympt HIV/VL) | -0.24000 | 0.0797 | -3.012 | 0.0415 |
| HEALTHY - (DTH+) | 0.01511 | 0.0814 | 0.186 | 1.0000 |
| HEALTHY - HIV | 0.00152 | 0.0742 | 0.021 | 1.0000 |
| HEALTHY - RECOVERED VL | -0.03488 | 0.0903 | -0.386 | 0.9997 |
| HEALTHY - VL | -0.03711 | 0.0844 | -0.440 | 0.9995 |
| (AIDS/VL) - (Asympt HIV/VL) | -0.14183 | 0.0644 | -2.202 | 0.2940 |
| (AIDS/VL) - (DTH+) | 0.11328 | 0.0667 | 1.699 | 0.6170 |
| (AIDS/VL) - HIV | 0.09969 | 0.0576 | 1.729 | 0.5961 |
| (AIDS/VL) - RECOVERED VL | 0.06329 | 0.0772 | 0.820 | 0.9830 |
| (AIDS/VL) - VL | 0.06107 | 0.0702 | 0.870 | 0.9770 |
| (Asympt HIV/VL) - (DTH+) | 0.25511 | 0.0705 | 3.619 | 0.0055 |
| (Asympt HIV/VL) - HIV | 0.24153 | 0.0620 | 3.896 | 0.0019 |
| (Asympt HIV/VL) - RECOVERED VL | 0.20512 | 0.0805 | 2.549 | 0.1420 |
| (Asympt HIV/VL) - VL | 0.20290 | 0.0738 | 2.750 | 0.0863 |
| (DTH+) - HIV | -0.01359 | 0.0642 | -0.212 | 1.0000 |
| (DTH+) - RECOVERED VL | -0.04999 | 0.0822 | -0.608 | 0.9966 |
| (DTH+) - VL | -0.05222 | 0.0757 | -0.690 | 0.9932 |
| HIV - RECOVERED VL | -0.03641 | 0.0751 | -0.485 | 0.9990 |
| HIV - VL | -0.03863 | 0.0679 | -0.569 | 0.9976 |
| RECOVERED VL - VL | -0.00222 | 0.0851 | -0.026 | 1.0000 |
