# Supplementary material for: T-cell activation and senescence in asymptomatic HIV/Leishmania infantum co-infection
Source: PLoS Negl Trop Dis. 2025 Mar 17;19(3):e0012848. doi: 10.1371/journal.pntd.0012848 (PMC11964262; doi:10.1371/journal.pntd.0012848)
Supplement: S8 Table — (DOCX) [file pntd.0012848.s010.docx]

| **Table S8. Pairwise comparisons of the marginal means of the percent of CD3+CD4+PD1+ proportion via beta regression model fit** | | | | |
| --- | --- | --- | --- | --- |
|  | Estimate | Std. Error | z-value | p-value |
| HEALTHY - (AIDS/VL) | -0.04168 | 0.0430 | -0.968 | 0.9607 |
| HEALTHY - (Asympt HIV/VL) | 0.00251 | 0.0425 | 0.059 | 1.0000 |
| HEALTHY - (DTH+) | 0.00532 | 0.0431 | 0.123 | 1.0000 |
| HEALTHY - HIV | 0.03827 | 0.0372 | 1.029 | 0.9475 |
| HEALTHY - RECOVERED VL | -0.10597 | 0.0593 | -1.787 | 0.5567 |
| HEALTHY - VL | -0.03243 | 0.0480 | -0.676 | 0.9939 |
| (AIDS/VL) - (Asympt HIV/VL) | 0.04419 | 0.0371 | 1.191 | 0.8978 |
| (AIDS/VL) - (DTH+) | 0.04700 | 0.0378 | 1.244 | 0.8767 |
| (AIDS/VL) - HIV | 0.07996 | 0.0309 | 2.587 | 0.1297 |
| (AIDS/VL) - RECOVERED VL | -0.06429 | 0.0556 | -1.157 | 0.9099 |
| (AIDS/VL) - VL | 0.00925 | 0.0433 | 0.214 | 1.0000 |
| (Asympt HIV/VL) - (DTH+) | 0.00280 | 0.0371 | 0.076 | 1.0000 |
| (Asympt HIV/VL) - HIV | 0.03576 | 0.0301 | 1.187 | 0.8994 |
| (Asympt HIV/VL) - RECOVERED VL | -0.10849 | 0.0551 | -1.968 | 0.4355 |
| (Asympt HIV/VL) - VL | -0.03494 | 0.0427 | -0.818 | 0.9833 |
| (DTH+) - HIV | 0.03296 | 0.0309 | 1.065 | 0.9383 |
| (DTH+) - RECOVERED VL | -0.11129 | 0.0556 | -2.002 | 0.4132 |
| (DTH+) - VL | -0.03774 | 0.0433 | -0.871 | 0.9768 |
| HIV - RECOVERED VL | -0.14425 | 0.0512 | -2.819 | 0.0717 |
| HIV - VL | -0.07070 | 0.0375 | -1.887 | 0.4892 |
| RECOVERED VL - VL | 0.07355 | 0.0595 | 1.237 | 0.8799 |
|  |  |  |  |  |
