# Supplementary material for: T-cell activation and senescence in asymptomatic HIV/Leishmania infantum co-infection
Source: PLoS Negl Trop Dis. 2025 Mar 17;19(3):e0012848. doi: 10.1371/journal.pntd.0012848 (PMC11964262; doi:10.1371/journal.pntd.0012848)
Supplement: S10 Table — (DOCX) [file pntd.0012848.s012.docx]

**Table S10. PCA loading for each analyzed variables**

| Variable | PC1 | PC2 | PC3 |
| --- | --- | --- | --- |
| rOD SLA | 0,6211 | -0,519 | 0,471 |
| rOD rK39 | 0,742 | -0,476 | 0,269 |
| CD3CD4CD38HLADR | 0,596 | 0,034 | -0,667 |
| CD3CD8CD38HLADR | 0,716 | -0,178 | -0,601 |
| CD3CD4PD1 | 0,634 | -0,004 | 0,357 |
| CD3CD8PD1 | 0,594 | 0,451 | -0,060 |
| CD3CD4CD57 | 0,249 | 0,724 | 0,337 |
| CD3CD8CD57 | 0,532 | 0,632 | 0,117 |
