# Supplementary material for: T-cell activation and senescence in asymptomatic HIV/Leishmania infantum co-infection
Source: PLoS Negl Trop Dis. 2025 Mar 17;19(3):e0012848. doi: 10.1371/journal.pntd.0012848 (PMC11964262; doi:10.1371/journal.pntd.0012848)
Supplement: S11 Table — (DOCX) [file pntd.0012848.s013.docx]

**Table S11. Distribution of individuals into K-means cluster groups**

| Group | AIDS/VL | Asympt HIV/Leish | DTH | HIV | RECVL | UC | VL |
| --- | --- | --- | --- | --- | --- | --- | --- |
| 1 | 2 | 0 | 0 | 0 | 1 | 0 | 3 |
| 2 | 3 | 2 | 9 | 14 | 4 | 6 | 2 |
| 3 | 6 | 2 | 0 | 0 | 0 | 0 | 2 |
| 4 | 2 | 6 | 0 | 2 | 1 | 0 | 1 |
