# Supplementary material for: T-cell activation and senescence in asymptomatic HIV/Leishmania infantum co-infection
Source: PLoS Negl Trop Dis. 2025 Mar 17;19(3):e0012848. doi: 10.1371/journal.pntd.0012848 (PMC11964262; doi:10.1371/journal.pntd.0012848)
Supplement: Attachment — Submitted filename: Mendes_AGUIAR_RESPONSE_REVIEWERS_LETTER_2024_R1_Aug_8.docx [file pntd.0012848.s016.docx]

Editor’s comments

Response to

Johan Van Weyenbergh

Academic Editor
PLOS Neglected Tropical Diseases

Susan Madison-Antenucci
Section Editor
PLOS Neglected Tropical Diseases

Dear Editor,

We thank for the review. We are sending below responses to the comments, in italic, which are written below them.

Sincerely,

Selma Jeronimo

Response to the Editor

*Thank you very much for submitting your manuscript "T-cell activation, senescence, and exhaustion in asymptomatic HIV/Leishmania infantum co-infection" for consideration at PLOS Neglected Tropical Diseases.*

*As with all papers reviewed by the journal, your manuscript was reviewed by members of the editorial board and by several independent reviewers. In light of the reviews (below this email), we would like to invite the resubmission of a significantly-revised version that takes into account the reviewers' comments*.

**R. Thank you for considering our manuscript and for the feedback by the reviewers. We revised it carefully and addressed all questions raised by the reviewers.**

[1] A letter containing a detailed list of your responses to the review comments and a description of the changes you have made in the manuscript. Please note while forming your response, if your article is accepted, you may have the opportunity to make the peer review history publicly available. The record will include editor decision letters (with reviews) and your responses to reviewer comments. If eligible, we will contact you to opt in or out.

**R. We addressed all comments raised by the reviewers.**

[2] Two versions of the revised manuscript: one with either highlights or tracked changes denoting where the text has been changed; the other a clean version (uploaded as the manuscript file).

**R. A clean and track change files were up-loaded, thank you**

**Comments Reviewer 2:**

*1. This manuscript characterized the prevalence of Leishmania infantum antibodies and delayed type hypsersensitivity reactions in a cross-sectional study of 1,372 people living with HIV infection in NE Brazil between 2014 and 2016. As visceral leishmaniasis (VL) is a reportable disease, VL cases occurring between 2014 and 2018 were cross-indexed with the cohort to identify cases. 69 subjects with HIV and/or L. infantum from the cross-sectional study were recruited for additional immunological evaluations. As described in other reports, the prevalence of infection with L. infantum was high based on antibody testing (ELISA against two antigens) and DTH responses. Different clinical outcomes of L. infantum (asymptomatic infection in HIV+ and HIV- subjects, VL with AIDS, symptomatic VL, recovered VL (following treatment), DTH+ with no history of VL, or healthy control. Flow cytometry studies examined immune activation (CD38+/HLADR+), senescence (CD57+), and exhaustion (PD-1)markers in CD4+ and CD8+ T cells.*

*The VL follow up in the cross-sectional cohort found that a positive ELISA was associated with a 2.27 relative risk of developing AIDS/VL over a relatively short period of follow up (< 4 years). This is important information that suggests that L. infantum serologic testing should be strongly considered in people living with HIV in endemic regions. More information regarding the group recruited for immunological studies is needed and the definition of the groups should be clarified.*

*It is stated the 69 subjects were “drawn from the cross-sectional study” (line 169), thus one would assume all are HIV+. However, there are 6 HIV negative control subjects, and 17 with VL that appear to not have HIV infection (8 VL and 9 recovered VL).*

*Were these individual’s part of the original cross-sectional study?*

**R. We apologize for not making the text clearer and we now carefully rewrote the information regarding the populations recruited for this study in the Methods section (pages 9-10) and presented more clearly their description in Table 1.**

**In summary, the groups included:**

**1. A cross sectional study of people with HIV who resided in the Natal area, a city of about 800 thousand people, that has been endemic for visceral leishmaniasis since the early 1990’s was performed** (JERONIMO et al., 1994; LIMA et al., 2017). **A serological survey for Leishmania infection was first performed in people with a prior diagnosis of HIV (1,372 people) between 2014-2016. From this population, a subgroup whose leishmania infection status was either positive or negative was later studied to assess immunological markers associated with T cell senescence, activation, and exhaustion. These people were either anti-leishmania positive or negative (page 9).**

**2. A retrospective analysis of the state of Rio Grande do Norte dataset composed of subjects who developed VL between 2014-2018 was performed to estimate the magnitude of AIDS-VL. In this group of AIDS/VL, we were able to identify subjects who were previously tested for Leishmania infection in our first Leishmania serological survey of subjects living with HIV, with these data it was possible to estimate the relative risk of developing VL in HIV subjects, page 9.**

**3. The symptomatic VL population were people immunocompetent (no HIV infection) who were being treated for VL in the Giselda Trigueiro Hospital and were part of a study of determinants of developing leishmaniasis.**

**4. The recovered VL and DTH+ people were drawn from a previous study in which immunocompetent individuals with VL and family members were studied. Those had previously been described** (Braz et al., 2002; I. D. Lima et al., 2012; JERONIMO, S. M. *et al.*, 2004; JERONIMO, S. M. B. *et al.*, 2007)**. They were screened for evidences of anti-leishmania antibodies and tested for their DTH responses against Leishmania antigens (DTH, Montenegro Test). Of importance, subjects with symptomatic VL do not mount a positive DTH response; they actually mount a proper response (induration greater than 5 mm) only after clinical recovery** (MILES et al., 2005)**.**

**5. The Healthy Control/uninfected controls were immunocompetent people with no prior history of *Leishmania infantum* infection.**

*2. The study lacks important information on HIV infection and treatment that may greatly influence the activation marker data interpretation. Specifically, AIDS/VL had uncontrolled HIV RNA with the known association of high viral load and reduced CD4+ T cell counts, increased activation, senescence and increased exhaustion markers. Since these activation markers are elevated in the VL and recovered VL groups (presumably HIV-negative), it is logical to assume that VL enhances the chronic immune activation associated with HIV infection.*

*Since immune activation also increases HIV viral load, and HIV viral load is inversely related to CD4 counts, it is possible that the VL is partially responsible for the worsened HIV markers suggesting a synergistic relationship, although the number of subjects is low and the power to dissect this relationship is limited.*

*The assumption by the authors that the high HIV viral load was due to poor antiretroviral therapy (ART) adherence. Specifically, how do the authors know that this is due to poor adherence versus newly diagnosed HIV infection? More information about the timing of ART in these individuals and how it relates to the immunologic results is needed to interpret these data.*

*If HIV+ people with VL present for care before or shortly after they receive ART, this confounds all activation measurements as there are HIV-mediated effects and immune reconstitution effects that may contribute to the activation, senescence and exhaustion results.*

**R. Unfortunately prior to 2013, treatment with ART in Brazil were started when subjects living with HIV had a CD4+ below 350 cells. This was changed with a protocol from the Brazilian Minister of Health which started to be implemented in 2013, when subjects who were diagnosed with HIV were promptly started with ART. However, the majority of the recruited subjects in this study had their HIV diagnosis prior to 2013 and their prior clinical history was not always possible to ascertain.**

**We added this information in the method section and addressed the limitations of this current study since these people living with HIV then did not have the opportunity that newly diagnosed HIV have. Our study population reported in this manuscript was drawn mostly from people without timely and adequately use of ART.**

**We are currently conducting a study which was started in 2021 (Bezerra et al, manuscript in preparation) and addressed exactly the questions raised by the reviewer 2, but with adherence to therapy right after first diagnosis of HIV infection.**

*In summary, this manuscript provides data on the risk of VL in an HIV cohort based on retrospective reportable disease data. The paper further describes immunological evaluations in a subgroup of this cohort.* ***Better definition of the group studied, and description of the HIV therapy in this group is needed to interpret the T cell activation/senescence/exhaustion findings.***

***R. We have addressed these questions in the method section, and also in the discussion.***

**Reviewer's Responses to Questions**

*Key Review Criteria Required for Acceptance?*

*As you describe the new analyses required for acceptance, please consider the following

Methods
- Are the objectives of the study clearly articulated with a clear testable hypothesis stated?
- Is the study design appropriate to address the stated objectives?
- Is the population clearly described and appropriate for the hypothesis being tested?
- Is the sample size sufficient to ensure adequate power to address the hypothesis being tested?
- Were correct statistical analysis used to support conclusions?
- Are there concerns about ethical or regulatory requirements being met?*

*Reviewer #1: (No Response*

*Reviewer #2: (No Response)*

***Reviewer #3: - Are the objectives of the study clearly articulated with a clear testable hypothesis stated?***

*The hypothesis was that people with coinfection with HIV and L. infantum would have altered T cell activation which would increase risk of visceral leishmaniasis. The authors should specify coinfection relative to what – presumably should be relative to L. infantum mono-infection. The presence of altered T cell activation could be (and was) easily tested but whether this increased activation would increase risk of visceral leishmaniasis is not easy to test.*

**R. We agree with the reviewer. Unfortunately, the data reported herein were prior to subjects living with HIV were promptly started on ART, as described above. As documented in the literature, subjects living with HIV without ART, tended to evolve with comorbidity (cancer and co-infections) and were more likely to develop diseases such as tuberculosis, toxoplasmosis and neoplastic disorders. However, there was not then electronic charts available and some clinical information were lost. The hypothesis of the risk of symptomatic disease with a second infection was well known and although the population was small (the ones who developed VL) it was enough to estimate the risk. This risk has entirely changed with the onset of prompt ART therapy. We addressed the reviewer’s concern as limitation of this current study in the discussion section.**

***- Is the study design appropriate to address the stated objectives?***

*The study design is appropriate to test the first part of the hypothesis but not the second part.*

*For the second part, the authors have compared immune profiles of participants with asymptomatic Leishmania infection plus HIV with those of symptomatic Leishmania plus HIV. However, it seems that this was a cross-sectional rather than longitudinal analysis so no causality can be attributed to the findings.*

*To answer the question whether T cell activation influences the development of leishmaniasis, you would really have to do a longitudinal analysis of the T cell immunology of people with HIV and asymptomatic leishmaniasis and compare the immunology of those who develop symptomatic leishmaniasis versus those who don’t.*

*This would have to be a large study considering that only 2.4% of participants with asymptomatic coinfection developed symptoms over three years. Even this would not be definitive – you would then need to design a trial where you somehow specifically reduced T cell activation to see whether this had an effect on development of symptomatic leishmaniasis. This would also have to be a very large study.*

**R. We agree with the reviewer’s comment which actually addressed the changing epidemiology of visceral leishmaniasis and people living with HIV in Brazil. This is the current scenario in Brazil; VL used to be a disease of rural areas, but as the country became urban, outbreaks occurred in the periurban areas (Jeronimo et al., 1994). With this urban scenario was possible to determine infection and outcome of *Leishmania infantum* infection** (JERONIMO et al., 2004)**. Parallel to this scenario, social changes in Brazil started in 1994,** (Andrade et al., 2009) **which led to a better family health program with increase in vaccination coverage** (LIMA et al., 2017, 2018) **for children. Based on previous studies performed in Northeast of Brazil, the risk of VL development for people once infected with Leishmania was shown to be 1 in six infected subjects developed VL (Badaro et al, 1986; Evans et al, 1992). Currently, more resistance to developing VL after *L. infantum* infection is being observed. In addition, disease has moved to a young adult age** (LIMA et al., 2017)**(Lima et al, 2019).**

**We added symptomatic and recovered VL and asymptomatic Leishmania infection (people who did not develop VL) groups, because of the knowledge that people with symptomatic VL, if they remain immunocompetent, have long last immunity, with hardly any documentation of relapse. Similarly, people with asymptomatic infection mounted a cellular response (Leishmania DTH positive subjects) have also long-lasting immunity. The reversibility in the patterns of exhaustion, senescence and activation of T cell responses have been described by several groups in both humans and dogs of VL, but there is reversal for humans; this was the reason for studying recovered VL. However, the reviewer is right; HIV induces activation, exhaustion and senescence (Figures 3 and 4), but Leishmania infection may worsen it. Potentially, this is the reason of more relapse and severity in people with HIV. We revised in the discussion these findings in the context of the literature and the ongoing research.**

*Considering the difficulty in funding such large studies, and the presumed paucity of evidence in this field, the authors have done a cross-sectional analysis which at least supports their hypothesis even if it cannot confirm it. The authors should highlight these issues and limitations in their discussion.*

**R. We addressed these issues in the discussion section. The reviewer’s is correct and it is even harder currently to carry such a study since improvement in care has occurred; which is a good thing, to see the risk of VL in both immunocompetent people and people living with HIV. Adherence to ART made a difference in the lives of these people and they must be empowered and increase their own care.**

*One other limitation is the variable rate of HIV viraemia and adherence to ART in the study population as seen in figure 2 and table 4. This is likely a major confounder for the association between T cell activation and development of asymptomatic and symptomatic leishmaniasis. The authors did not measure antigen-specific T cell activation so this may have been non-specific as a consequence of viraemia rather than a response to L. infantum.*

**R. The reviewer is right, but the fact that Leishmania alone can lead to the pattern seen induced by HIV suggests that the combination of the two infections worsen the outcome. In a previous study of first few cases of AIDS/VL relapse and death were greater than subjects with VL alone** (NASCIMENTO et al., 2011)**.**

*Indeed, the authors highlight that antigen-specific T cell activation is minimal in active leishmaniasis. They argue that asymptomatic coinfected participants likely had antigen-specific T cell responses (lines 450-454) but this is on the basis of weak evidence and they should acknowledge that it may alternatively just relate to reduced levels of HIV suppression. Indeed, they found that adherence to ART was highly predictive of the clinical leishmania status in people living with HIV. The authors should report any correlation between HIV viral load and T cell activation which they stated they performed in lines 361-364.*

**R. All of the data on correlations are described in the manuscript. We found correlation between exhausted TCD8 cells and presence of HIV infection. Viral load was not correlated with any parameters.**

*This distinction matters because it affects interpretation of the results. It may simply be that maintenance of suppressive ART is all that is needed to prevent either asymptomatic or symptomatic leishmania infection. The authors argue that there may be a role for anti-leishmania treatment in asymptomatic coinfected individuals but they need to acknowledge that without antigen-specific testing they cannot determine whether the T cell activation observed in these individuals is helping to control infection. They should also acknowledge that, with rates of development of symptomatic leishmaniasis of only 2.4% within three years amongst those with initially asymptomatic coinfection, it would be necessary through longitudinal studies to identify risk factors within this group for development of active disease in order to stratify which asymptomatic coinfected patients might benefit from anti-leishmania treatment. This could then be tested in a clinical trial.*

***R. We agree with the reviewer and have added a sentence to the end of the Discussion that reads “Given the relatively low incidence of VL observed here, further studies are needed to identify risk factors for active disease that could inform a clinical trial of anti-leishmanial treatment in a more targeted population, such as persons with low or falling CD4+ T counts or uncontrolled viral loads.” We have started the careful follow up of a population of 829 people living with HIV on ART. Based on this, we can propose to the Brazilian Minister of Health such a clinical trial.***

***- Is the population clearly described and appropriate for the hypothesis being tested?***

*It isn’t entirely clear where some of the 69 participants for the immunologic analyses came from. They can’t all have come from the cross-sectional analysis, as stated in lines 154-155, because the cross-sectional study was only of people living with HIV who presumably were all asymptomatic of leishmaniasis when they had their initial serology. Were the AIDS/VL participants all initially part of the cross-sectional study? This should be stated. Also, where did the HIV-negative participants come from?*

**R. We now better defined the populations in the method section (pages 9-10) and Table 1. The AIDS/VL participants were not part of the cross-sectional study but rather derived from the Notifiable Diseases Information System as detailed in the text.**

*Please note the correct term is 'people living with HIV', not 'HIV positive subjects', ie refer to the person first and the disease second. This is globally accepted as appropriate terminology as requested by numerous advocacy groups.*

**R. We appreciate the input and have replaced HIV positive subjects by people living with HIV.**

***- Is the sample size sufficient to ensure adequate power to address the hypothesis being tested?*** *Yes it clearly was because the results were significant. Trying to assess this prospectively is nothing more than a guessing game if there is no preliminary data – this paper now provides preliminary data for future studies (at least in this region).*

**R. Thank you. It actually led us to perform the prospective study which we are finishing the analysis.**

- ***Were correct statistical analysis used to support conclusions?***

I believe so. However, it appears that no statistician was used to analyse results; the authors instead used GraphPad Prism and ‘omni calculator’ available online. It is probably worth getting a statistician to review the analytical methods.

**R. We had the data reviewed by a statistician who is now a co-author of the manuscript and we added supplementary tables which were the results of the new analysis.**

- ***Are there concerns about ethical or regulatory requirements being met?***

*The authors should state whether the consent form was signed before any study-related activities were commenced. They should also state whether the study was registered online prior to commencement.*

**R. The protocol was reviewed and approved by the Ethical Committee. The study was not registered online (e.g. clinicaltrials.gov) as this is not a requirement for observational studies. Informed consent was obtained, and the consent form was signed by the participant or their legal guardian prior to the performance of any study-related activities. Exemption of consent was obtained for those from the cross-sectional study when contact was not possible.**

**Results**
*-Does the analysis presented match the analysis plan?
-Are the results clearly and completely presented?
-Are the figures (Tables, Images) of sufficient quality for clarity?<*

*Reviewer #1: (No Response)*

*Reviewer #2: (No Response)*

**Reviewer #3:**

**- Does the analysis presented match the analysis plan?**

*Not entirely; the authors should mention the correlation analyses – which ones were prospectively decided on and how the correlation analyses were performed*.

**R.** **We have added to the Statistical analysis section that we evaluated strengths of relationships between two continuous variables Spearman’s correlation coefficient (rho). Correlations were assessed based on *a priori* plans.**

**- Are the results clearly and completely presented?**

*I don’t understand why tables S1 to S4 were included when this information is already in figures 1 to 4 (apart from the non-significant p values – which can just be omitted as it is clear they are non-significant, ie p > 0.05).*

***R. We removed former tables S1, S2 and S4, since the figures were informative and the tables show the median, and table S3 is renamed as table S1.***

*There are some discrepancies – eg table S2 AIDS/VL vs asymptomatic HIV/leish, table S3 CD57 vs recovered VL and PD-1+ vs DTH+ (both for CD4+ T cells) – please check whether the table or the figure is correct.*

***R. We checked all statistics and results. Table 2 was removed and the results are in the text.***

*I also don’t understand the purpose of table 2 – all of this information is already in the text.*

**R. We removed table 2 and just left the information in the text.**

Line 220 – what does ‘high titers’ mean? Suggest changing this to ‘positive titers’ if appropriate.

**R. We reworded the text, removed high titers, and indicated the results for the relative risk of developing VL for those with prior anti-leishmania antibodies.**

Line 227 – should reword to say relative risk for development of VL in participants living with HIV with versus without positive leishmania serology (ie specify what this is relative to).

**R. Thank you. We reworded it.**

Line 239 – suggest using a different acronym to CS – not clear what this stands for (presumably control subjects) – suggest UC for uninfected controls and spell this out.

**R. Thank you. We reworded and replaced CS for UC and we spelled out as uninfected control.**

Was there any significant difference between groups in gender balance?

**R. As we and others published before** (JERONIMO et al., 2004; LIMA et al., 2018, 2012)**, the overall risk of developing VL in an immunocompetent population is greater for males, although infection occurs similarly for all independent of age or sex. However, in this work we did not design it to study the sex questions within people living with HIV; but the analysis showed no gender difference.**

Was any age / gender matching performed between groups selected for immunologic analysis?

**R: We did not initially match for age or gender; however, the statistical analysis did not show differences with respect to age or gender.**

What do the horizontal bars indicate on the graphs? This should be stated in the legends. HIV appears twice in the x axis for figure 2A.

**R: Thank you for observation. We changed it in the figure.**
Description of some of the results is far too wordy and difficult to read – especially lines 311 to 328. This doesn’t add anything over just looking at the figure which is far easier to interpret. I suggest simplifying the description significantly, focusing on overall and interesting results and leaving out most of the non-significant findings unless they are interesting/surprising. It can also be useful to introduce these multiple findings by starting with what you expected (and why) and then reporting what you actually found.

**R: Thank you for suggestion. We rewrote this part of the text.**

Why were some correlation analyses interspersed with the other results when other correlation analyses were reported in the final paragraph? I would suggest putting the correlation analyses all together in the final paragraph.

**R: Thank you for suggestion. We placed all the correlations together in the last paragraph.**

In lines 336-7 the authors state that asymptomatic HIV/Leish had more senescent CD8+ T cells than HIV but they appear to contradict this in the next sentence; I think the wording of that next sentence needs to be changed. Again, I would suggest focusing on the significant results rather than the non-significant ones.

**R: Thank you for suggestion. We rewrote the results.**

Line 341 – a positive correlation should have a positive r value.

**R: Thank you for observation. We corrected it.**

Line 351 – beware of saying ‘similar proportions’ when they are not similar at all – they are just not statistically different.

**R: Thank you for observation. We corrected it.**

Line 359 - ? missed the word rk39?

**R: Thank you for this observation. No, this sentence was rewritten and the word “and” wasn’t deleted. The sentence was rewritten and moved to the last paragraph.**

Line 362 – was this really a multivariable analysis (ie multiple regression) or just multiple separate correlation analyses? The term multivariable analysis implies multiple regression.

**R: Thank you for observation. We rewrite the last paragrapher. Multivariable analysis and Correlations were indicated in sentences.**

- Are the figures (Tables, Images) of sufficient quality for clarity?

Yes.

--------------------

<b>Conclusions</b></br></br>
-Are the conclusions supported by the data presented?</br>
-Are the limitations of analysis clearly described?</br>
-Do the authors discuss how these data can be helpful to advance our understanding of the topic under study?</br>
-Is public health relevance addressed?</br></br>

Reviewer #1: (No Response)

Reviewer #2: (No Response)

Reviewer #3: - Are the conclusions supported by the data presented?

Not entirely. Line 450 – overreach to say that it is ‘probable’ that asymptomatic HIV/Leish subjects can mount a specific Th1 response to SLA simply based on similar frequencies of activated CD4+ T cells to DTH and lower levels of CD4+ T cell activation than AIDS/VL participants.

**R: Thank you for your observation, we rewrote this sentence.**

Lines 480-483 – overreach to say ‘suggesting’ asymptomatic pts could be in the beginning stages of progression to functional impairment. The authors could perhaps say ‘raising the possibility’. Most participants didn't develop VL. It could also be that senescence / exhaustion reflects increased HIV or Leish antigen exposure or gut barrier permeability and could be adaptive in preventing disease and that other factors are responsible for developing symptoms. The authors should mention this possibility.

**R: Thank you for suggestion. We rewrote this part of the text.**

Line 484-485 – participants are mostly on ART in this group so it isn’t accurate to say that they seem to be mostly controlling both infections.

**R: Not all participant take ART correctly, as showed in the results. Moreover, Asymptomatic HIV-Leishmania subjects seem to controlling both infection because they had similar viral load levels as HIV subjects and did not development VL symptoms like AIDS/VL patients.**

Lines 487-490 – a much more likely explanation is that most participants in the AIDS/VL group weren’t taking ART. I suggest starting with this and then saying that asymptomatic VL may have had an additional impact on T cell exhaustion although one can’t exclude that this was also due to being less likely on ART than those who weren’t infected with L. infantum.

**R: Thank you for your suggestion. We rewrote the sentence to better present our hypothesis.**

Line 493 – the word ‘can’ should be changed to ‘may be able to’ and clearly defined – to refer to T cell exhaustion and senescence rather than CD4+ T cell decline.

**R: Thank you for suggestion. Suggestion accepted.**

Line 499 – ‘suggest’ is an overreach. I suggest changing this to ‘raise the possibility that’.

**R: Thank you for suggestion. Suggestion accepted.**

Lines 502-503 - This paper provides no evidence to support pre-emptive therapy. I suggest concluding that longitudinal studies are required to determine whether elevation in T cell senescence and exhaustion predicts VL even in those with suppressed virus.

**R: Thank you for observation. We rewrote the last sentence.**

Line 446 - Reference 5 shows that antigen stimulation did not UPREGULATE activation markers above baseline (compare figures 1 and 2 in reference 5), not that the cells didn’t EXPRESS activation markers following antigen stimulation.

**R: Thank you for suggestion. Suggestion accepted.**

Line 456 – This line appears to conflict with lines 445-446; the authors should explain what they mean more clearly.

**R: Thank you for observation. We rewrote the last sentence.**

- Are the limitations of analysis clearly described?

No, not at all. The authors should highlight that causality cannot be inferred from a cross-sectional study and that this paper is hypothesis-generating only. Furthermore, the lack of even gender/age matching makes it hard to interpret whether differences between groups could be due to these factors or other potential confounders. The lack of antigen-specific testing makes it hard to know whether elevations in immune activation are a response to parasite antigen or not. The difference between groups in ART adherence is likely a major confounder. They need to highlight which investigations would be required next to take things further.

**R: Thank you for all appointments about limitations. We wrote the discussion section including these statements**

- Do the authors discuss how these data can be helpful to advance our understanding of the topic under study?

No. They draw tentative conclusions without adequately acknowledging alternative more likely explanations or drawing attention to the limitations of the study.

**R: In this study we showed new data about asymptomatic HIV/Leishmania co-infected subjects. We revised and rewrote the discussion section and include the study limitations. Here, we observed high relative risk to development AIDS/VL by asymptomatic HIV/Leishmania subjects, and it could be influence by poor adherence to ART and previous history of chronic infections. Now we are following up a new Asymptomatic HIV/Leishmania cohort. All participants have had good adherence to ART, the absence of past chronic infections, and no one has developed AIDS/VL after three years.**

- Is public health relevance addressed?

Not really. They highlight the surprisingly high frequency of seropositivity amongst people living with HIV and the changing epidemic with HIV entering rural areas and L. infantum entering urban areas but don’t discuss how this could be addressed from a public health perspective (eg long-sleeved clothing, insect repellent, bed nets).

**R: The manuscript points out differences in immune status of asymptomatic HIV-Leishmania subjects versus others HIV groups. These observations shown in the manuscript also indicated high relative risk for the development of AIDS-VL among the Asymptomatic HIV-Leish subjects, and raise the question of how we can manage these subjects in a public health system.**

--------------------

<b>Editorial and Data Presentation Modifications?</b></br><br/>
Use  this section for editorial suggestions as well as relatively minor modifications of existing data that would enhance clarity. If the only modifications needed are minor and/or editorial, you may wish to recommend “Minor Revision” or “Accept”.

Reviewer #1: (No Response)

Reviewer #2: (No Response)

Reviewer #3: Please see first and third paragraphs under “Are the results clearly and completely presented?” in the results section above.

**R: We rewrote parts of the Results section for clarity.**

--------------------

<b>Summary and General Comments</b></br></br>
Use  this section to provide overall comments, discuss strengths/weaknesses of the study, novelty, significance, general execution and scholarship. You may also include additional comments for the author, including concerns about dual publication, research ethics, or publication ethics. If requesting major revision, please articulate the new experiments that are needed.

Reviewer #1: Visceral leishmaniasis can be fatal, and co-infection with HIV increases the risk of death. Therefore, understanding the impact of HIV on responses to VL (and vice-versa) is important. This manuscript aims to study changes in the phenotype of T cells in co-infected patients, and the authors have access to a large number of patients to analyze the different functions of T cells. This could lead to a greater understanding of the immune response to these infections, and how they impact diseases. However, the analysis is limited, the description of the results needs to be improved, and the data representation could be more friendly to the reader. In addition, the interpretation of the data is overstated, and the results are not contextualized with the literature.

Specific comments:

1.      There is no explanation of what is T cell senescence and exhaustion and why the authors chose these functions and those markers (CD57 and PD1) to characterize them.

them.

**R: In results, we introduced paragraphs with some explanation of function of CD57 and PD-1, and then we showed our results about a specific marker mentioned before.**

2.      How do the authors know that AIDS/VL subjects have VL? Is DTH the criteria? This is important because then it means that these patients have defects in antibody production, but T cell responses are unaffected. If correct, how do the authors interpret those findings? The criteria should be clearly explained in the methods section.

**R: The groups’ characterization is present in table 1. Symptomatic VL characterization is described above table 1: “The VL status was confirmed by either parasitological findings in the bone marrow aspirate or by presence of anti- *L. infantum* antibodies and with clinical findings consistent with the disease”.**

3.      No interpretation of the findings is provided, making it difficult to read the manuscript. For example: “a negative correlation was observed between activated CD4+T cells and CD4+ counts”. What is the authors’ interpretation of this finding? This lack of interpretation and contextualizing of the results with the literature is widespread throughout the manuscript.

**R: The Results section was rewritten and negative correlations were excluded.**

4.      What is the relative risk of developing AIDS/VL compared to other infectious diseases?

**R: This rate can be estimated prior to introduction to ART in which tuberculosis, toxoplasmosis and others infectious disease** (NASCIMENTO et al., 2011)**. However, with the introduction of ART these rates of other infectious diseases may vary since it depends on the prevalence of the particular infectious agent. For instance, PPD or IGRA for tuberculosis estimate that 64% of HIV subjects had IGRA-TB positive test** (KROON et al., 2020)**. Toxoplasma serology was positive in 73% of new HIV cases, independently of CD4 counts** (DUTTA; MEHTA; INGOLE, 2020)**. However, we did not focus on the other diseases since we did not have assays run able to detect infection. We do have data on the number of diseases, but not infection. We added comment in the discussion section**

5.      PD-1 is a T cell activation marker. The expression of PD-1 alone does not mean that T cells are exhausted. How do the authors interpret their findings, given that they only looked for PD-1 expression?

**R: PD-1 is known to be an exhaustion marker since it suppresses T cell activation and effector functions after chronic antigen stimulation.**

6.      There is no consensus in the literature on whether CD4 T cells become exhausted because there is no clear demonstration that they have the loss of effector functions like CD8 T cells do. The authors make no comments on this.

**R: In HIV infection, CD4+PD1+ cells harbor HIV and lose effector capacity. When PD-1 is blocked, CD4+PD1+ T cells reverse HIV latency, suggesting these cells are functionally active** (JUBEL et al., 2020)**.**

7.      In lines 345-346, the authors state: “Remarkably, there were few other significant differences in CD4+ T cell exhaustion markers between the groups”. Why not explain what those small differences are and what they mean? Also, why some of the data is shown as a table instead of a figure?

**R: In figure 2 we showed all significant differences between groups. We also changed this part of text, describing better this topic.**

8.      What is the meaning of the “weak association” between exhausted and senescent CD8 T cells and CD8 counts (lines 367-368).

**R: weak association is related with low r^2^, however, the correlation is significant and rewrote it.**

Minor comments:

1.      Describe the acronyms in figure/table legend. CS, for example, needs to be described.

Reviewer #2: (No Response)

**R: We described all acronyms in table 1 and in figures 1 and 2.**

Reviewer #3: Please see comments already provided. Additionally, the authors should comment on whether a study of this nature has been published before (ie whether these are novel findings). Overall there are a large number of issues that should be addressed before this paper could be considered for publication.

**R: It is the first study analyzing immune status of Asymptomatic HIV-Leishmania subjects. Other authors showed variables percentage of Asymptomatic HIV-Leishmania subjects, using serology to Leishmania or Leishmania DNA by PCR. However, none of them describe or analyze activation, senescence and exhaustion in this group of subjects. It is important to highlight that this group needs more attention paid to ART adherence, since we found a high relative risk forthe development of AIDS-VL in this group with low ART adherence.**

--------------------

PLOS authors have the option to publish the peer review history of their article (<a href="<https://journals.plos.org/plosntds/s/editorial-and-peer-review-process#loc-peer-review-history>" target="_blank">what does this mean?</a>). If published, this will include your full peer review and any attached files.<br><br>

If you choose “no”, your identity will remain anonymous but your review may still be made public.<br><br>

<b>Do you want your identity to be public for this peer review?</b> For information about this choice, including consent withdrawal, please see our <a href="<https://www.plos.org/privacy-policy>" target="_blank">Privacy Policy</a>.

Reviewer #1: No

Reviewer #2: No

Reviewer #3: No

Referências

Andrade, I. G., Queiroz, J. W., Cabral, A. P., Lieberman, J. A., & Jeronimo, S. M. B. (2009). Improved sanitation and income are associated with decreased rates of hospitalization for diarrhoea in Brazilian infants. *Transactions of the Royal Society of Tropical Medicine and Hygiene*, *103*(5), 506–511. https://doi.org/10.1016/j.trstmh.2008.12.017

Braz, R. F. S., Nascimento, E. T., Martins, D. R. A., Wilson, M. E., Pearson, R. D., Reed, S. G., & Jeronimo, S. M. B. (2002). The sensitivity and specificity of Leishmania chagasi recombinant K39 antigen in the diagnosis of American visceral leishmaniasis and in differentiating active from subclinical infection. *American Journal of Tropical Medicine and Hygiene*, *67*(4), 344–348. https://doi.org/10.4269/ajtmh.2002.67.344

Lima, Á. L. M., de Lima, I. D., Coutinho, J. F. V., de Sousa, Ú. P. S. T., Rodrigues, M. A. G., Wilson, M. E., Pearson, R. D., Queiroz, J. W., & Jerônimo, S. M. B. (2017). Changing epidemiology of visceral leishmaniasis in northeastern Brazil: A 25-year follow-up of an urban outbreak. *Transactions of the Royal Society of Tropical Medicine and Hygiene*, *111*(10), 440–447. https://doi.org/10.1093/trstmh/trx080

DUTTA, Anuja; MEHTA, Preeti; INGOLE, Nayana. Seroprevalence of Toxoplasma gondii in newly diagnosed HIV seropositive patients. **The Indian Journal of Medical Research**, *[S. l.]*, v. 152, n. 5, p. 515, 2020. DOI: 10.4103/IJMR.IJMR_110_19. Disponível em: /pmc/articles/PMC8157894/. Acesso em: 5 ago. 2024.

JERONIMO, S. M. et al. An emerging peri-urban pattern of infection with Leishmania chagasi, the protozoan causing visceral leishmaniasis in northeast Brazil. **Scand J Infect Dis**, *[S. l.]*, v. 36, n. 6–7, p. 443–449, 2004. Disponível em: http://www.ncbi.nlm.nih.gov/pubmed/15307565.

JERONIMO, S. M. B. et al. Genes at human chromosome 5q31.1 regulate delayed-type hypersensitivity responses associated with Leishmania chagasi infection. **Genes and Immunity**, *[S. l.]*, v. 8, n. 7, p. 539–551, 2007. DOI: 10.1038/sj.gene.6364422.

JERONIMO, Selma M. B. et al. An urban outbreak of visceral leishmaniasis in Natal, Brazil. **Transactions of the Royal Society of Tropical Medicine and Hygiene**, *[S. l.]*, v. 88, n. 4, p. 386–388, 1994. DOI: http://dx.doi.org/10.1016/0035-9203(94)90393-X. Disponível em: http://www.sciencedirect.com/science/article/pii/003592039490393X.

JUBEL, Jil M.; BARBATI, Zachary R.; BURGER, Christof; WIRTZ, Dieter C.; SCHILDBERG, Frank A. The Role of PD-1 in Acute and Chronic Infection. **Frontiers in Immunology**, *[S. l.]*, v. 11, p. 524474, 2020. DOI: 10.3389/FIMMU.2020.00487/BIBTEX. Disponível em: www.frontiersin.org. Acesso em: 5 ago. 2024.

KROON, Elouise E. et al. An observational study identifying highly tuberculosis-exposed, HIV-1-positive but persistently TB, tuberculin and IGRA negative persons with M. tuberculosis specific antibodies in Cape Town, South Africa. **EBioMedicine**, *[S. l.]*, v. 61, 2020. DOI: 10.1016/J.EBIOM.2020.103053. Disponível em: https://pubmed.ncbi.nlm.nih.gov/33038764/. Acesso em: 5 ago. 2024.

LIMA, Ádila L. M.; DE LIMA, Iraci D.; COUTINHO, José F. V.; DE SOUSA, Úrsula P. S. T.; RODRIGUES, Marcos A. G.; WILSON, Mary E.; PEARSON, Richard D.; QUEIROZ, José W.; JERÔNIMO, Selma M. B. Changing epidemiology of visceral leishmaniasis in northeastern Brazil: A 25-year follow-up of an urban outbreak. **Transactions of the Royal Society of Tropical Medicine and Hygiene**, *[S. l.]*, v. 111, n. 10, p. 440–447, 2017. DOI: 10.1093/trstmh/trx080.

LIMA, I. D.; LIMA, A. L. M.; MENDES-AGUIAR, C. O.; COUTINHO, J. F. V; WILSON, M. E.; PEARSON, R. D.; QUEIROZ, J. W.; JERONIMO, S. M. B. Changing demographics of visceral leishmaniasis in northeast Brazil: Lessons for the future. **PLoS Negl Trop Dis**, *[S. l.]*, v. 12, n. 3, p. e0006164, 2018. DOI: 10.1371/journal.pntd.0006164. Disponível em: http://www.ncbi.nlm.nih.gov/pubmed/29509765.

LIMA, Iraci D. et al. Leishmania infantum chagasi in Northeastern Brazil: Asymptomatic infection at the urban perimeter. **American Journal of Tropical Medicine and Hygiene**, *[S. l.]*, v. 86, n. 1, p. 99–107, 2012. DOI: 10.4269/ajtmh.2012.10-0492.

MILES, Suzanne A.; CONRAD, Sean M.; ALVES, Renata G.; JERONIMO, Selma M. B.; MOSSER, David M. A role for IgG immune complexes during infection with the intracellular pathogen Leishmania. **Journal of Experimental Medicine**, *[S. l.]*, v. 201, n. 5, p. 747–754, 2005. DOI: 10.1084/jem.20041470.

NASCIMENTO, E. T.; MOURA, M. L. N.; QUEIROZ, J. W.; BARROSO, A. W.; ARAUJO, A. F.; REGO, E. F.; WILSON, M. E.; PEARSON, R. D.; JERONIMO, S. M. The emergence of concurrent HIV-1/AIDS and visceral leishmaniasis in Northeast Brazil. **Transactions of the Royal Society of Tropical Medicine and Hygiene**, *[S. l.]*, v. 105, n. 5, p. 298–300, 2011. DOI: 10.1016/j.trstmh.2011.01.006. Disponível em: http://dx.doi.org/10.1016/j.trstmh.2011.01.006.
