# Supplementary material for: T-cell activation and senescence in asymptomatic HIV/Leishmania infantum co-infection
Source: PLoS Negl Trop Dis. 2025 Mar 17;19(3):e0012848. doi: 10.1371/journal.pntd.0012848 (PMC11964262; doi:10.1371/journal.pntd.0012848)
Supplement: Attachment — Submitted filename: Mendes_aguiar_Response_Reviewers_2.docx [file pntd.0012848.s017.docx]

Responses to Reviewer’s Questions

Key Review Criteria Required for Acceptance?

*As you describe the new analyses required for acceptance, please consider the following:*

**Methods**
-Are the objectives of the study clearly articulated with a clear testable hypothesis stated?

-Is the study design appropriate to address the stated objectives

-Is the population clearly described and appropriate for the hypothesis being tested?
-Is the sample size sufficient to ensure adequate power to address the hypothesis being tested?
-Were correct statistical analysis used to support conclusions?

-Are there concerns about ethical or regulatory requirements being met?

Reviewer #1: (No Response)

Reviewer #2: *The authors have addressed the concerns raised in my first review whenever possible, and the manuscript is strengthened by these changes*.

Reviewer #3: *The wording of this redraft is much improved. However, there is still a lot of work* to do.

**Abstract**
-       Lines 39-40 – *altered T cell activation relative to whom?*

*The authors still haven’t specified this and need to do so as otherwise this sentence is rather meaningless. Based on the content of the paper, the answer should be relative to PLHIV without Leishmaniasis. This part of the hypothesis was addressed in the paper but the second part was not (“that would increase the risk of VL”) – this part can only be evaluated with an interventional study. I would say something like “We hypothesised that, relative to those with HIV alone, people with co-infection would have altered T cell activation which could impact on the risk on the risk of development of VL”.*

**R: Thank you for noticing that we had claimed the wrong comparison group. We have rewritten the hypothesis to state that HIV/VL coinfected persons are more likely to develop symptoms of VL than individuals with *L. infantum* infection alone. We corrected this in the text.**

-       I suggest not referring to asymptomatic HIV/Leish, symptomatic VL, recovered VL etc in the abstract as these terms are meaningless to readers. I suggest the authors rather describe what they mean – eg Immune status of 69 participants was evaluated and comparisons made between those with HIV without, with latent or with active Leishmania infection and those without HIV but with active or resolved Leishmania or T cell hypersensitivity to Leishmania antigen. There are multiple instances where the authors have referred to asymptomatic HIV/Leish etc which should be changed.

**R: Thank you for pointing out the unclarity of the abstract. We rewrote the section describing the groups, and tried to make it clear what we were referring to. We have used the words latent and active to define asymptomatic and symptomatic *L. infantum* infection, although we have retained the term “asymptomatic” to be consistent with other literature that uses this term.**

-       The authors should include the confidence intervals around the relative risk of 2.2 and a p value given that this was one of the key questions of interest identified in the hypothesis; I presume this is not significant given that the confidence interval crosses 1. They should also explain who the comparator group is in this sentence.

**R: Thank you for suggestion. We inserted 95% , RR and p-value in the abstract. The reviewer is correct that the difference is not significant (p=0.07)**

- “Poor adherence to antiretroviral therapy… was associated with development of AIDS/VL” - the authors should specify which group they are referring to (?HIV alone or HIV with positive Leish serology or both).

**R: Thank you for observation, we were referring** asymptomatic HIV/Leish. We rewrote the sentence.

**Author summary**

-       PLWH – it is requested by people living with HIV to avoid turning them into an acronym. When an acronym is necessary due to word limits, the correct one is PLHIV (without a W). However, wherever possible, it is best to write “people living with HIV” rather than PLHIV. There are multiple instances of PLWH throughout the paper.

**R: The first version of this manuscript, we wrote the expression “people living with HIV”. The acronym PLWH was suggested by former reviewer. We agree with this reviewer that PLHIV is more descriptive, so we have switched to this terminology in the current version of the manuscript.**

-       Referring to asymptomatic HIV/Leish, symptomatic VL, recovered VL etc in the author summary is fairly meaningless to readers who haven’t read the paper yet – I would suggest not using these terms in the author summary but simply describing the comparisons which are most relevant, eg markers of ___ were higher in people living with HIV with positive Leishmania serology compared to those with HIV but negative Leishmania serology and were similar to those with HIV / active Leishmania coinfection.

**R: Thank you for suggestion. We rewrote the abstract, and we have attempted to make the study groups clear.**

**Introduction**
-       The authors should mention somewhere in the introduction that the immunologic profile of people living with HIV with positive serology for L. infantum has not previously been described.

**R: Thank you for suggestion. We add in the introduction your suggestion.**
-       Line 94 - IFAB should be I-FABP

**R: Thank you for suggestion. We rewrote this.**

-       Line 94 – “consequently” should be “indicative of”

**R: Thank you for suggestion, we substituted the term.**

-       Line 121 – “indeed” should be “furthermore” as the sentence following is about CD8+ T cells rather than CD4+ T cells in the previous sentence.

**R: Thank you for suggestion.**

-       Line 129 – The authors should specify that symptomatic VL, recovered VL and DTH+ were all people without HIV.

**R: Thank you for suggestion. We rewrote this part of the text.**

-       Line 133 and 135 – “influenced” should be “was associated with” – the authors cannot make causative assertions based on cross-sectional data even if the literature supporting such causation is strong.

**R: Thank you for suggestion. We made the change.**

-       Line 134 – suggest adding “latent or active” before “L. infantum”

**R: Thank you for suggestion. We rewrote this part of the text.**

-       Line 135 – suggest changing “or” to “and”

**R: Thank you for suggestion, we rewrote the sentence.**

-       Line 137 – The authors should specify which group had a higher risk of active Leishmaniasis / HIV coinfection in comparison to which group; otherwise this sentence doesn’t have much meaning.

**R: Thank you for observation. We rewrote this part of the text.**

**Methods**
-       Line 166 – “from which” rather than “which”

**R: Thank you for suggestion. Change accepted.**

-       In table 1, suggest specifying in the recovered VL and DTH+ groups that these participants were immunocompetent without HIV as it is possible to be immunocompetent with HIV (if diagnosed and commenced on treatment early).

**R: Thank you for suggestion. Change accepted.**

-       Line 236 – suggest adding “before any study-related procedures” after “guardian” (the authors verified this in their response to my comments but didn’t add this to the manuscript)

**R: Thank you for suggestion. Change accepted.**

-       Regarding exemption of consent, does this just refer to those whose only data is in the original cross-sectional study, ie who didn’t have repeat bloods? Might be worth clarifying this in the manuscript.

**R: Thank you for suggestion. We rewrote this part of the text.**

--------------------
Results
-Does the analysis presented match the analysis plan?

-Are the results clearly and completely presented?

-Are the figures (Tables, Images) of sufficient quality for clarity?

Reviewer #1: (No Response)

Reviewer #2: The authors have addressed the concerns raised in my first review whenever possible, and the manuscript is strengthened by these changes.

**Reviewer #3**

**Results**

-       Line 256 – please add a p value; I suspect this will be > 0.05 given that the 95% CI crosses 1.

**R: Thank you for suggestion. We rewrote this part of the text**

-       Line 260 – Most subjects in each group were male – incorrect – should be eg “four of the seven groups had a male predominance while the remainder were more evenly balanced”.

**R: Thank you for suggestion. We rewrote this part of the text.**

-       Line 310 – specify that VL, recovered VL and DTH+ are without HIV (same for figure 2)

**R: Thank you for suggestion. We rewrote this part of the text**

-       Lines 323 to 339 – this paragraph is still very challenging to read as there are so many comparisons described many of which are not of interest. I would suggest starting with an overall comment (“groups a, b and c had the highest frequencies of x overall with no significant differences found between them”) and then focusing on the relevant/interesting findings (“all three groups had higher frequencies of x than d, e and f for both CD4+ and CD8+ T cells”). The same should be done with the subsequent paragraphs to make it easier to read and understand.

**R: Thank you for suggestion. We rewrote this part of the text**

-       Figure 1 – why do the p values differ from tables S4 and S5? Why have two different methods been used to do the statistical analysis? I would suggest just one method; otherwise it looks like the authors are fishing for positive results. The authors should also review the horizontal significance bars over the figures – some comparisons are clearly significant (as shown in the tables, eg UC vs AIDS/VL for CD8+CD38+HLA-DR+) but there is no horizontal bar showing this in the figure.

**R:**

**- Tables S4 and S5 are different analysis; Table S4 compared marginal** **means in activated CD4 T cells, whereas Table S5 compared marginal means in activated CD8 T cells.**

**-In figure 1, p values of horizontal significance bars were generated by GraphPad prism 8.0, and we used a non-parametric Kruskal-Whallis test with Uncorrected Dunn´s test, because not all data passed in the normality test Moreover, the statistical analysis shown in Supplementary Tables presented parametric generalized linear model and with the fit of the GLM followed by pair-wise comparisons, using Wald-type tests. In the first manuscript version, only non-parametric Kruskal-Wallis test with Uncorrected Dunn´s test were shown.**

**- horizontal significance bar to UC vs AIDS/VL for CD8+CD38+HLA-DR+ was included.**

-       Figure 2 – the same issues should be reviewed as for Figure 1 – I suggest the authors use one statistical approach for each comparison rather than two. The p values should then be fixed and the horizontal bars should be reviewed.

**R: In figure 2, p value of horizontal significance bars were generated by GraphPad prism 8.0, and we used a non-parametric Kruskal-Wallis test with Uncorrected Dunn´s test. Moreover, the statistical analysis shown in Supplementary Tables presented parametric generalized linear model and with the fit of the GLM followed by pair-wise comparisons, using Wald-type tests. In the first manuscript version, only non-parametric Kruskal-Wallis test with Uncorrected Dunn´s test were shown.**

We also performed Principal component analysis (PCA) to tease out how the concomitant infections by L. infantum and HIV influenced the immune response. All 69 subjects included with the immunological parameters were considered. The parameters are presented in Figures 1 and 2, and Table 2.

-       Lines 326-328 – this sentence implies that this was a longitudinal analysis; I suggest the authors reword this to say x was lower than y rather than imply that something decreased after recovery.

**R: Thank you for suggestion. We rewrote this part of the text.**

-       Lines 340-342 – I suggesting putting this the other way around, ie CD57 was higher in these groups than this group. The subsequent sentence is a repeat sentence.

**R: Thank you for suggestion. We rewrote this part of the text.**

-       Line 358 – “senescent” should be “exhausted”

**R: Thank you for suggestion. Change accepted.**

-       Line 363 – The authors should include the multivariable analysis results in a table in the supplement including odds ratios and 95% CIs.

**R: New tables for multivariable analysis were included in supplementary tables with 95% CI and p values, but odds ratios were not included, when the dependent variable was a count.**

-       Line 369 – The authors need to specify in the manuscript which Spearman correlations were prespecified and performed. The results of the correlations should be shown in the supplement including graphs for significant associations.
**R: Thank you for suggestion. Figures are included in supplemental material.**

--------------------

Conclusions
-Are the conclusions supported by the data presented?

-Are the limitations of analysis clearly described?

-Do the authors discuss how these data can be helpful to advance our understanding of the topic under study?

-Is public health relevance addressed?

Reviewer #1: (No Response)

Reviewer #2: The authors have addressed the concerns raised in my first review whenever possible, and the manuscript is strengthened by these changes.

Reviewer #3:

Discussion

-       Line 412 – “Thus, in HIV+ population” – the authors should say “in people living with HIV” but more importantly this sentence is a non-sequitur - the previous sentence was talking about active infection in immunocompetent individuals and this sentence is talking about latent infection in people living with HIV so the word “thus” is not justified. The authors should acknowledge in the manuscript that sensitivity of serology is unknown in this population and explain why they didn't use DTH as the screening test.

**R: Thank you for suggestion. We rewrote this part of the text.**

**- The use of DTH was discontinued by the Brazilian Healthy Ministry. DTH+ subjects studied here were drawn from a previous study in which immunocompetent individuals with VL and family members were studied. Those had previously been described (Braz et al., 2002; I. D. Lima et al., 2012; JERONIMO, S. M. *et al.*, 2004; JERONIMO, S. M. B. *et al.*, 2007).**

**- In this work was used two different antigens to screen positive serology. rK39 is a recombinant antigen with high specificity and sensitivity to discriminate active VL infection. Our serology results show similar sensitivity to other data in the literature. Depending on the geographic area, the prevalence of positive serology can vary from 3-25 (**Colomba C, Saporito L, Vitale F, Reale S, Vitale G, Casuccio A, et al. Cryptic Leishmania infantum infection in Italian HIV infected patients. BMC Infect Dis. 2009;9. doi:10.1186/1471-2334-9-199).

Rezaei Z, Sarkari B, Dehghani M, Layegh Gigloo A, Afrashteh M. High frequency of subclinical Leishmania infection among HIV-infected patients living in the endemic areas of visceral leishmaniasis in Fars province, southern Iran. Parasitol Res. 2018;117: 2591–2595. doi:10.1007/s00436-018-5949-9

Ena J, Pasquau F, Del Mar LόPez-Perezagua M, Martinez-Peinado C, Arjona F. Screening for subclinical leishmania infection in HIV-infected patients living in eastern Spain. Pathog Glob Health. 2014;108: 356–361. doi:10.1179/2047773214Y.0000000164

-       Line 433 – “recurring” should I think be “relapsing”

**R: Thank you for suggestion. Change accepted.**

-       Line 440 – suggest adding a line saying that it would have been helpful to have an additional group which was positive Leishmania serology without HIV to help evaluate these questions.

**R: Thank you for suggestion. We rewrote this part of the text and include this limitation.**

-       Lines 456-459 – This is a non-sequitur - what is the evidence that overall CD4+ T cell activation predicts risk of development of activated Leishmania in coinfected individuals? The only evidence that asymptomatic HIV/Leish subjects can probably control infection is that they are asymptomatic but this clearly is not necessarily a permanent state. I suggest deleting this sentence.

**R: Thank you for suggestion. Change accepted.**

-       Line 459-460 – “apparently influenced” should be changed to “was associated with” as no causality can be inferred by cross-sectional data.

**R: Thank you for suggestion. Change accepted.**

-       Line 463 – “indicates the presence of Leishmania infantum infection increase” should be changed to “suggest the presence of Leishmania infantum infection may increase” – again causality cannot be inferred.

**R: Thank you for suggestion. Change accepted.**

-       Line 466 – IFAB should be I-FABP

**R: Thank you for suggestion. Change accepted.**

-       Line 477 – “influenced” should be “was associated with”

**R: Thank you for suggestion. Change accepted.**

-       Line 485-486 – “CD4+ proportions” should be “CD4+ T cell counts”. I would probably leave this part of the sentence out actually as the convention is to look at the frequency of cell subsets as a proportion of the parent population rather than absolute values in blood.

**R: Thank you for suggestion. Change accepted.**

-       Line 496 – “we hypothesize that with progression of asymptomatic to symptomatic VL” is repeated.

**R: Thank you for observation. We excluded repeated text.**

-       Line 497 – “could” – what could? This makes no sense – the subject is missing. If the subject is “progression of asymptomatic to symptomatic VL” then the word “with” should be removed.

**R: Thank you for suggestion. Change accepted.**

-       Line 504 – “immunosuppression” – no need to invoke immunosuppression here – increasing activation of the immune system would be sufficient to amplify disease progression in the absence of ART.

**R: Thank you for suggestion. Change accepted.**

-       Lines 510-511 – the authors should specify what else they could have used in addition to serology (eg ?DTH). Using PCR to detect latent disease makes no sense – I would presume that a positive PCR would indicate active infection.

**R: We included DTH test in this part of the sentence. A positive PCR test can indicate the presence of live or dead parasite by detection of kDNA.**

-       Lines 514-515 – This is a non-sequitur - why do the authors decide to call PLHIV with positive serology asymptomatic based on symptomatic pts being less likely to have positive serology? They are asymptomatic based on being asymptomatic. The authors should reword this to make it logical or delete it.

**R: Thank you for observation. We changed this part of the text.**

-       An additional line should be inserted in the limitations paragraph saying that another limitation was that people with positive Leishmania serology but without HIV were not included and could have helped to evaluate whether HIV and Leishmania were having synergistic effects.

**R: Thank you for suggestion. We rewrote this part of the text and include this limitation.**

-       Line 519 – the authors should expand on the difference between groups in ART adherence and how this may entirely account for the difference between HIV and asymptomatic HIV/Leish groups in activation / exhaustion / senescence findings.

**R: Thank you for suggestion. We rewrote this part of the text.**

-       Line 520 – “can lead to” should be “is associated with” – the authors can’t infer causality as stated in the previous paragraph!

**R: Thank you for suggestion. Change accepted.**

-       Line 522 – “temporary” – what are the authors basing this assertion on? This is a cross-sectional study. If there is longitudinal data supporting this statement then it should be referenced here.

**R: Thank you for suggestion. We rewrote this part of the text.**

-       Line 523 – “after treatment” – after what treatment? For HIV or for Leishmania?

**R: Thank you for suggestion. We rewrote this part of the text.**

-       Lines 529-532 – I know it was me who suggested this line but it is actually quite a weak way to end the article as the implication is that we should be focusing our efforts on working out which patients to treat for latent Leishmaniasis when in fact these patients should all be on ART for its mortality benefit based on international guidelines. I would suggest changing to “Longitudinal trials are needed to determine whether, in the setting of universal ART and sustained viral load suppression, T cell activation and exhaustion are seen in people living with HIV with positive serology for Leishmania and, if so, whether these immune parameters predict active VL despite suppressed virus”. This is, after all, what the authors are now doing.
**R: Thank you for suggestion. Change accepted.**

--------------------

Editorial and Data Presentation Modifications?

Use  this section for editorial suggestions as well as relatively minor modifications of existing data that would enhance clarity. If the only modifications needed are minor and/or editorial, you may wish to recommend “Minor Revision” or “Accept”.

Reviewer #1: (No Response)

Reviewer #2: None

Reviewer #3: (No Response)

--------------------

Summary and General Comments

***SELMA, THIS IS THE MOST DAMAGING COMMENTN THAT I SEE. CHANGES NEED TO BE MADE THROUGHOUT INDICATING WE STUDIED ONLY ONE MARKER OF EXHAUSTION AND WHAT IS OBSERVED IS REALLY A DIFFERENCE IN SURFACE PHENOTYPE. TO DRAW CONCLUSIONS ABOUT EXHAUSTION WE WOULD NEED TO DOCUMENT OTHER EXHAUSTION MARKERS ARE ALSO UP (LAG-3, TIM1 ETC) AND DO FUNCTIONAL STUDIES. WE ONLY SEE SURFACE MARKERS SUGGESTING CHANGES MIGHT BE BEGINNING***.
Use  this section to provide overall comments, discuss strengths/weaknesses of the study, novelty, significance, general execution and scholarship. You may also include additional comments for the author, including concerns about dual publication, research ethics, or publication ethics. If requesting major revision, please articulate the new experiments that are needed.

Reviewer #1: The authors did not satisfactorily address this reviewer's comments, particularly contextualizing their findings with the vast literature on exhaustion and senescence. It does not look like the authors understand the complex literature on exhaustion or failed to demonstrate it in their manuscript. For example, PD-1 is induced by TCR engagement, and, therefore, activated T cells will express PD-1 merely by seeing antigen. Long-term exposure to antigen will generate exhausted T cells. However, there is an array of inhibitory receptors, and PD-1 is just one of them. What creates a major issue is not using PD-1 as a marker of exhaustion, but it is that the authors failed to contextualize and address the caveats to their findings.  As exhaustion, and senescence are central components of the manuscript and part of the title of the paper, altering the manuscript was absolutely necessary and the authors failed to thoughtfully incorporate the criticism.

**R: We included sentences and literature of this issue and added a paragraph about exhaustion.**

Reviewer #2: The authors have addressed the concerns raised in my first review whenever possible, and the manuscript is strengthened by these changes.

Reviewer #3: The suggestions are outlined above. There is still a lot of rewording required and the authors should include the results of the multivariable and correlation analyses in the supplement.

--------------------

PLOS authors have the option to publish the peer review history of their article (<a href="<https://journals.plos.org/plosntds/s/editorial-and-peer-review-process#loc-peer-review-history>" target="_blank">what does this mean?</a>). If published, this will include your full peer review and any attached files.<br><br>

If you choose “no”, your identity will remain anonymous but your review may still be made public.<br><br>

<b>Do you want your identity to be public for this peer review?</b> For information about this choice, including consent withdrawal, please see our <a href="<https://www.plos.org/privacy-policy>" target="_blank">Privacy Policy</a>.

Reviewer #1: No

Reviewer #2: No

Reviewer #3: No
