# Supplementary material for: T-cell activation and senescence in asymptomatic HIV/Leishmania infantum co-infection
Source: PLoS Negl Trop Dis. 2025 Mar 17;19(3):e0012848. doi: 10.1371/journal.pntd.0012848 (PMC11964262; doi:10.1371/journal.pntd.0012848)
Supplement: Attachment — Submitted filename: response_reviewers_R3_final.docx [file pntd.0012848.s018.docx]

January 10, 2025.

Dear Editor,

Please find below our responses to the reviewers. We also added the two manuscript files (track changes and clean versions) and the revised tables and figures.

We appreciate this final revision.

Sincerely,

Selma M.B. Jeronimo

**Additional Editor Comments :**

The editors and reviewers have extensively discussed the manuscript in its current and previous versions, hence the delay in formulating a decision.

Two major points remain crucial: ***overstressing the exhaustion message and adding a PCA analysis*** that does not answer the scientific hypothesis.

*1. Reviewer #1 rightfully critiqued the overselling of the “exhaustion” message based on a single marker PD-1, which is also a marker or early T-cell activation, and specifically asked to discuss this extensively. The authors only reply with a short paragraph, which is poorly formulated. They also do not mention using a single marker (PD-1) as a major limitation, as requested, and did not remove the claim of T-cell exhaustion from the title, abstract or any other part of the manuscript. Considering PD-1 is also a marker of T-cell activation, this is also in keeping with the authors’ working hypothesis in the abstract: “We hypothesized that, relative to those with HIV alone, people with co-infection would have altered T cell activation which could impact on the risk of VL.”*

R. The exhaustion term was removed from the title and abstract and the other parts of the manuscript and we revised carefully the manuscript to avoid overstressing the exhaustion message. We maintained the PCA, but included also PCA3 and PCA4.

2. The authors claim that PCA analysis discriminates between Leishmania-infected and non-infected individuals.

First, this is unsurprising since anti-Leishmania serology was one of the few parameters included to define PCs.

Second, the authors did not perform a cluster analysis and/or test cluster quality (silhouette score or similar), due to the spreading of patients it seems unlikely that clusters can be discriminated.

Third, the authors only analyzed PC1 and PC2, although they do not explain 75% of the variance, and they do not show the possible differential loadings of humoral and cellular parameters on PC1 and PC2 (or PC3-PC4).

**R. We considered the other component analysis and we included the Silhouette plot with 3 PCAs, with 4 groups with an average width of 0.61 (Figure S2) and two supplementary Tables (S10 and S11).**

*Since the total review process has been extremely long, and the manuscript has scientific merit in the editors’ opinion, we would like to give the authors the chance to address these two points thoroughly in a (final) revision.*

**Journal Requirements:**

1) Please ensure that the CRediT author contributions listed for every co-author are completed accurately and in full.
   At this stage, the following Authors/Authors require contributions:

Carolina de Oliveira Mendes Aguiar,

Selma M.B. Jeronimo.

Please ensure that the full contributions of each author are acknowledged in the "Add/Edit/Remove Authors" section of our submission form.
The list of CRediT author contributions may be found here: <https://journals.plos.org/plosntds/s/authorship#loc-author-contributions>

R. We reviewed and added their roles.

2) Tables should not be uploaded as individual files. Please remove these files and include the Tables in your manuscript file as editable, cell-based objects. For more information about how to format tables, see our guidelines:
https://journals.plos.org/plosntds/s/tables

R. We included the tables in the manuscript.

3) We have noticed that you have a list of Supporting Information legends in your manuscript. However, there are no corresponding files uploaded to the submission.

Please upload the supplementary tables as separate files with the item type 'Supporting Information'.

R. We uploaded the tables and supplementary figures as Supporting Information.

4) We notice that your supplementary figure is uploaded with the file type 'Figure'. Please amend the file type to 'Supporting Information'. Please ensure that each Supporting Information file has a legend listed in the manuscript after the references list.

R. We upoloaded the supplementary figures as Supporting information.

5) Thank you for stating that "All data are presented and the results will be available." We strongly recommend all authors decide on a **data sharing plan** before acceptance, as the process can be lengthy and hold up publication timelines. Please note that, though access restrictions are acceptable now, your entire data will need to be made freely accessible if your manuscript is accepted for publication. This policy applies to all data except where public deposition would breach compliance with the protocol approved by your research ethics board. If you are unable to adhere to our open data policy, please kindly revise your statement to explain your reasoning and we will seek the editor's input on an exemption. Please be assured that, once you have provided your new statement, the assessment of your exemption will not hold up the peer review process.

**R. The data sharing file was submitted**

*6) Please amend your detailed Financial Disclosure statement in the online submission form. This is published with the article. It must therefore be completed in full sentences and contain the exact wording you wish to be published.
1) State the initials, alongside each funding source, of each author to receive each grant. For example: "This work was supported by the National Institutes of Health (####### to AM; ###### to CJ) and the National Science Foundation (###### to AM)."
2) State what role the funders took in the study. If the funders had no role in your study, please state: "The funders had no role in study design, data collection and analysis, decision to publish, or preparation of the manuscript."*

R. We revised the text in the electronic submission and it reads now:

The work was supported from a grant from the CNPq (universal 406076/2021-9) awarded to SMBJ and National Institute of Science and Technology of Tropical Diseases (INCT-DT, 465229/2014-0) awarded to EMC and SMBJ. The funders had no role in study design, data collection and analysis, decision to publish, or preparation of the manuscript.

7) Your current Financial Disclosure states, "Brazilian Research Council - CNPQ - universal 406076/2021-9 and National Institute of Science and Technology of Tropical Diseases (INCT-DT, 465229/2014-0)".However, your funding information on the submission form indicates only one funder. Please ensure that the funders and grant numbers match between the Financial Disclosure field and the Funding Information tab in your submission form. Note that the funders must be provided in the same order in both places as well.                                             . 
Please indicate by return email the full and correct funding information for your study and confirm the order in which funding contributions should appear. Please be sure to indicate whether the funders played any role in the study design, data collection and analysis, decision to publish, or preparation of the manuscript.

R. We revised the funders and added the sentence related to the role of the funders.

**Reviewers' Comments:**

[NOTE: If reviewer comments were submitted as an attachment file, they will be attached to this email and accessible via the submission site. Please log into your account, locate the manuscript record, and check for the action link "View Attachments". If this link does not appear, there are no attachment files.]

**Figure resubmission:**

While revising your submission, please upload your figure files to the Preflight Analysis and Conversion Engine (PACE) digital diagnostic tool, https://pacev2.apexcovantage.com/. PACE helps ensure that figures meet PLOS requirements. To use PACE, you must first register as a user. Registration is free. Then, login and navigate to the UPLOAD tab, where you will find detailed instructions on how to use the tool. If you encounter any issues or have any questions when using PACE, please email PLOS at figures@plos.org. Please note that Supporting Information files do not need this step. If there are other versions of figure files still present in your submission file inventory at resubmission, please replace them with the PACE-processed versions.

**R: All figures were reviewed by PACE.**

**Reproducibility:**

To enhance the reproducibility of your results, we recommend that authors of applicable studies deposit laboratory protocols in protocols.io, where a protocol can be assigned its own identifier (DOI) such that it can be cited independently in the future. Additionally, PLOS ONE offers an option to publish peer-reviewed clinical study protocols. Read more information on sharing protocols at https://plos.org/protocols?utm_medium=editorial-email&utm_source=authorletters&utm_campaign=protocols
